# Tweets don’t vote – Twitter discourse from Wales and England during Brexit

**DOI:** 10.3389/fsoc.2023.1176732

**Published:** 2023-11-16

**Authors:** Larissa Peixoto Gomes

**Affiliations:** ^1^Wales Governance Centre, Cardiff University, Wales, United Kingdom; ^2^Institute for Globally Distributed Open Research and Education (IGDORE), Gothenburg, Sweden

**Keywords:** Brexit, Wales, England, Twitter, immigration, race, attitudes

## Abstract

The Welsh vote for “leave” in the Brexit referendum surprised some academics and analysts due to its strong preference for Labor and its close financial ties to the EU. It also brought up a debate about apparent differences in Welsh and English attitudes towards race, ethnicity, and migration, with the former often claiming to have a more positive stance regarding the presence of ethnic minorities and foreign nationalities. This paper proposes to analyze discourse posted on Twitter during June 2016, specifically targeting Wales and England with the aim to offer insight into the perceptions and beliefs of Welsh and English individuals on the platform and if attitudes on race, ethnicity, and migration played a significant role. Counterfactuals are checked with posts from the first few weeks of the refugee crisis in Afghanistan in 2021, the war on Ukraine, and the announcement of the Rwanda policy. The current discussion of Welsh national identity includes its claims as a “nation of sanctuary” and that understands oppression and marginalization. Thus, Welsh perspectives on Brexit become an interesting viewpoint to comprehending ethnic minorities and foreigners as it creates a possible conflict between the institutional discourse, cultural views, and perceived economic needs. In this context, this paper takes the view that Twitter is an area where individuals post their thoughts uninhibited, and where we can conduct an aggregate analysis of that public sentiment.

## 1 Introduction

This paper aims to discuss views on Brexit from England and Wales on Twitter.^1^ Specifically, the goal is to determine if English and Welsh tweeters used different discourses during Brexit and if those were specifically present for issues of race, immigration, and ethnicity.

Wales is often treated as “England and Wales”, rather than afforded unique analytical status, such as Scotland and Northern Ireland are. However, despite voting for Brexit by 52.5%, Wales is referred to as a Labor stronghold due to its electing a majority of Labor MPs for the House of Commons and its local authorities and maintaining a Labor government since devolution. This has led to views and policies that claim Wales to be more progressive and welcoming to ethnic minorities and foreigners in comparison to its neighbor.

Although Twitter data is not statistically representative of any population, it is exemplary of how conversations flow during major events. Studying the content of tweets has become a staple in public opinion research, aiding in analyses of information flow, fake news dispersion, and political opinion building (Jungherr, 2016; Almeida, 2017; Almeida et al., 2021).

Statistics of the population of Twitter are bit more difficult to dissect, as they are subject to much variation and little update from the site itself.^2^ Estimated from the last data made available by Twitter, in 2013, the Twitter is currently around 317 to 328 million, with likely 16 million being in the UK (LSE, n/d). In the UK, 95% households with at least one adult below 65 years of age have internet access; 70% of individuals with internet access use some form of social media (Office for National Statistics, 2020).

In Wales, 98% of households with at least one child have internet access, dropping to 87% when analyzing total households, with Powys the only local authority among the ten with lowest Digital Propensity Score^3^ (Office for National Statistics, 2021). Nevertheless, only areas in South Wales had DPI higher than 92.5%. Out of the local authorities with the highest DPI increase, 8 out of 10 are in Wales. Between the ages of 16 to 64, between 93 to 98% of people use the internet, depending on their employment status, with internet users accessing it daily (94%) or several times a day (74%) (National Survey for Wales, 2019). In Wales, 8 in 10 people with internet access use social media, with 24% accessing Twitter with any frequency, and 11% daily (Song et al., 2020).

By analyzing the rhetoric appearing during June 2016, I am able to identify how English and Welsh tweets are similar or different^4^ in their discourse, the connections made, the topics highlighted, the individuals mentioned. Using mainly the counterfactual of Ukraine, I demonstrate that the rhetoric changes when individuals feel their rights or privileges are threatened, but are more sympathetic when it is an abstract harm to others.

This paper contributes to research into the discourse in Wales and England during Brexit, with a particular view on Wales and its unique characteristics. Rather than consider the nation of Wales as a region of England, we can understand its peculiarities and needs and how its opinions are shaped. The complexity of the Brexit rhetoric and vote are briefly discussed, particularly its overall treatment as an electoral event between Conservatives and Labor rather than an outlet for various sentiments surrounding immigration, austerity, and lack of access to resources. Some comparison is made to topics that roused similar sentiments regarding immigration especially, namely the Afghan and Ukrainian refugee crises and the policy to send asylum seekers to Rwanda (“Rwanda policy”).

## 2 Literature review

The Brexit referendum occurred on 23 June 2016 and asked the simple question of British citizens: stay or leave the European Union? Many analyses have approached the topic from different perspectives, attempting to understand the breadth and impact of fake news, the lack of coherence with the Conservative Party, the lukewarm campaign by the Labor Party, and the intense polarization that created a seemingly perpetual ideological cleavage in British society.

Despite the vote being unified, that is, specific regions had no more no less say than other, much attention has been given to the differences in how the UK geographically divided itself in this vote. Scotland and Northern Ireland voted to remain in the EU, as did London, but Wales did not. This was a seemingly a surprise to some, as Wales has been a Labor stronghold for 100 years, electing a majority of Labor MPs to the House of Commons and having a Labor government since devolution, and largely depended on EU funding, leading to politicians and analysts believing it was a guaranteed “remain” overall. However, only five of its local authorities chose “remain”.

Both in the referendum itself and in the analyses, Brexit is often treated as common electoral event, rather than a unique experience that did not explicitly map on to typical party identification. Several analyses have attempted to align regions’ choice with their party allegiances, some looking for the explanation in the national UK Parliament vote and others in the European Parliament vote (Harris and Charlton, 2016; Becker et al., 2017). In Wales, this has meant looking at where Plaid Cymru is strongest and where UKIP won for the European Parliament and the proportional Senedd elections.

The Welsh Labor campaign for “remain” is considered to have been weak, as if a Labor vote equalled a “remain” vote and therefore a “sure thing” (Jones, 2016; Williamson, 2018; Mackay, 2020). Along with the referendum being scheduled five weeks after the Senedd elections, the campaign was unable to galvanize a base in support for “remain”. An apparent proximity between the Welsh elite and its general population is also thought to have given a general feeling that “remain” would ultimately win (Mackay, 2020).

On the other hand, the sentiment that Wales is a more tolerant nation has been pointed out as a “top-down” policy, rather than an actual cultural characteristic (Williams, 2015). Wales does not experience mass migration such as England, but there is evidence of racism against the ethnic minorities present there, both criminal (Williams and Tregidga, 2013) and every day, subtle racism (Goodman et al., 2014; Williams, 2015; Parker, 2018). These are often difficult to tease out, partly because police statistics and other studies are released or conducted as “England and Wales” and partly because evidence of subtle bias and racism is not only hard to identify, but also to obtain.

Nevertheless, as a country, Wales has positioned itself as “nation of sanctuary” and against stringent British migration policy (Welsh Government, 2019, 2020; Bernhardt, 2023). The official Welsh attitudes towards immigration follows their perception of their own invasion and persecution, an “othering” of the English that would then welcomes those looking for a home of their own (Williams, 2015; Bernhardt, 2023). This, however, is argued to be a foundational myth of the state, the establishment of a narrative that is then not fulfilled when we look at the upper echelons of Welsh society and its inclusion of ethnic minorities (Chaney, 2015; Evans et al., 2015) or, again, its everyday interactions (Goodman et al., 2014; Offord, 2016; Parker, 2018). While some regions in Wales are somewhat multicultural, particularly in the south (Evans et al., 2015; Mackay, 2020), Wales is predominantly white. Its industrial and mining history, along with other disputes with central British government, strongly account for its Labor predominance, which do not necessarily translate as support to the rest of its agenda.

A few analyses have been made regarding the “leave” vote overall and in Wales. It has been positively associated with age and negatively associated with education and socioeconomic status (Becker et al., 2017; Quintus, 2018; Alabrese et al., 2019). This, however, is an oversimplification of the reasons for the vote. These characteristics have thus been grouped into the concept of “left-behind”, which has also been questioned (Becker et al., 2017; Lee et al., 2018; Furlong, 2019; Martin et al., 2019) due to oversimplification. They neglect relevant factors and dynamics that led not only to “leave” winning, but winning in an upset in many regions. Feeling “left-behind”, either due to lack of access to services, poor quality of living, lack of access to consumer goods, is not the prerogative of any specific group and it necessary to also associate that feeling to someone else having access to your own detriment. Alternatively, given that the population of Wales is older, poorer, and has lower educational attainment than England, some were surprised that its vote for leave was so close (Mackay, 2020). Without a firm answer, the presence of English voters in Wales and this possible feeling of being “left-behind” and lack of “who to blame”, despite Wales’s alleged progressiveness are claimed as to why Brexit gained its majority. Areas expected to vote “leave” had also had higher turnout than expected – areas that are higher in deprivation (Becker et al., 2017; Gough, 2017). Importantly, research has neglected how and who different groups assign responsibility for the issues they face.

It follows that analyses on the rhetoric “getting Brexit done” show that Boris Johnson was able to capitalize on populist messaging reinforced place identification, propelling the connection of “left-behind” with an individual’s region. Those in the “geographies of discontent” looked forward to the promised levelling up investment and the protection of the NHS, while those with higher incomes were susceptible to the populist claims of cultural superiority (Cooper and Cooper, 2020). These themes and rhetoric are also associated with the referendum campaign, signaling keeping British money in British soil for British people, which resonated with those worse and better off. A repeated discourse of “self-determination” and “taking back control” was also reinforced in the “leave” campaign, generating a narrative of an oppressive regime against the UK, that a “leave” could then free the British people from some invisible oppression (Gherghina and O’Malley, 2019; Spencer and Oppermann, 2020). Combined with a feeling of loss of control in one’s personal life given a decline in quality of life, the Brexit referendum seemingly tapped into a feeling of “needing change” without knowing what it was. The “leave” campaign availed itself of a “David vs. Goliath” narrative, where a “leave” voter was David themselves, fighting against the oppression of the European Union and the European Parliament. This type of narrative was shown to be at least partially the reason for the success of “leave”, as it brought a cohesive argument to the public that resonated and generated conclusions from its original premise (oppression meant loss in funds to the EU which meant fewer funds for the NHS). The “remain” campaign was unable to weave together a cohesive and dense narrative that generated a conclusion of no change should occur (Spencer and Oppermann, 2020). These studies, however, are done for the country as a whole, either UK or England and Wales, not discerning the two nations as separate entities.

This study aims to contribute to the field by analyzing what was said in English and Welsh Twitter during the month of the referendum. This is relevant given the few studies on Wales, particularly on ground-level opinion and the possibility to view fresh data on the topic. Evidence has been shown that geographical boundaries matter in Twitter (Hedayatifar et al., 2020), and that Wales and England do appear as separate regions in the virtual space (Arthur and Williams, 2019; Hedayatifar et al., 2020). In addition to the specific geographic boundaries, vocabulary differences have also been found between Wales and regions in England (Arthur and Williams, 2019). While Twitter has been able to bring individuals from different areas together, it does not erase the need for local connection, camaraderie, and understanding (Hedayatifar et al., 2020).

## 3 Methods

Using the Twitter API and the academictwitteR package, data on four themes was collected, selecting key dates and tweets geotagged as United Kingdom. The themes and dates were, respectively^5^:

- Afghanistan refugee crisis: 01–31 August 2021
- Brexit: 01–30 June 2016
- Rwandan policy: 01–23 April 2022
- Ukraine war: 24 February – 24 March 2022

Collection on these was done based on a single word, “afghanistan”, “brexit”, “rwanda”, and “ukraine” (Gorodnichenko et al., 2021). A collection on non-related topics was conducted to verify the proportion of tweets between England and Wales and any possible bot interference. The tests demonstrated that regardless of topic, one can expect a rough proportion of 95% English tweets and 5% of Welsh tweets in a geotagged collection.^6^

In addition to Twitter’s own geotagging information, to ensure the highest amount of completion, data from the users’ description regarding their location was also used to increase the number of tweets in the dataset. While this does not ensure perfect data and may generate some coding errors, it does allow for those users that do not automatically input their location but do include it in their bios or profiles in any number of ways (for example, rather than a city location, users might feel more attached to their county or region; many Welsh users were found to add the Welsh location name rather or in conjunction with the English name). The process of manually including individuals’ named locations added between 0.5 to 1.5% of tweets to each dataset collect, and thus proved helpful in ensuring as many unique voices were added as possible.

The number of unique users for each revealed similar proportions for each theme, that is, roughly 95% English and 5% Welsh; in the Brexit-only tweets, there were 17,238 unique English users, and 977 Welsh users, 94.6 and 5.4%, respectively.

Due to the sizeable difference in tweets from English and Welsh sources, all clean-up and preparation was done separately to ensure English data did not interfere with the analysis of Welsh data. Consideration was given to randomly sample the English dataset to form a similarly sized corpus, but methodologically, as both datasets are the universe of tweets, it would not be a correct comparison.

Typical data clean-up was conducted, removing stop words, punctuation, and numbers, and setting words to lower case. Hashtags were removed, but @ symbols were kept as these differentiate between mentioning someone and tagging them. A collocation analyses yielded word sequences that were statistically significant or that had a qualitative relevance, such as “Queen and country” or “no recourse to public funds”. Expressions such as these have been joined and analyzed as a single entity. A number of “control words” were also set, from the top features of each document-feature matrix and sentiment words and expressions were also included. Lastly, the theme words “brexit”, “rwanda”, “afghanistan”, and “ukraine” were removed. This was done due to their high number of mentions which confounded the analysis – since they are the topics of discussion, it was irrelevant that they should be included. Finally, the dataset was trimmed using the keywords mentioned (detailed in the Appendix).

The data was cleaned and analyzed with the quanteda, tm, stm, and topicmodels packages (and additional support packages) for R, enabling a quantitative text analysis approach, sentiment analysis, and a targeted qualitative analysis of specific patterns in speech and rhetoric.

## 4 Results

**Figure 1:**
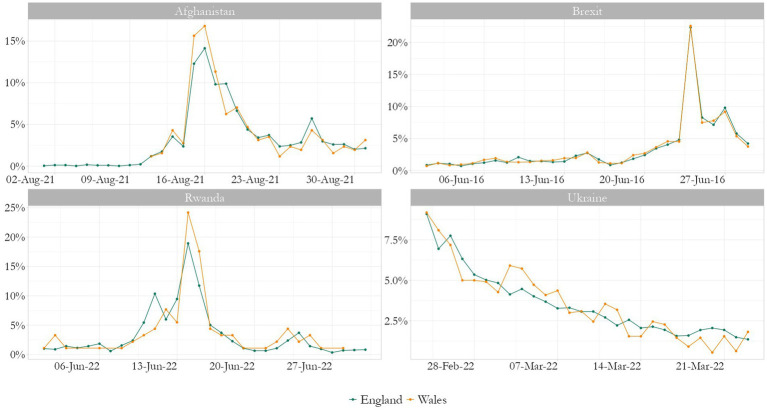
Relative frequency of tweets per nation, theme, and day. Source: the author, with data from the Twitter API.

Figure 1 shows the relative frequency of tweets per nation, theme, and day. There are clearly defined peaks and both nations tend to tweet the same volume at the same time. This corroborates findings about information dissemination on Twitter and what is, essentially, loss of interest in a topic. Although the Rwandan policy was brough up at different times of the year, the highest volume of tweets is in June 2022, specifically after the UK justice system decided on its legality. The untrimmed data shows the same relative frequency.

The first analysis is of keyness, which uses chi2 with a Yates’s correction to determine the relevance of a particular word or expression to a corpus. Keyness may be affected by the size of each group in the corpus, so I ran three analyses: one for a “brexit” corpus, with Wales and England being compared; and one for each nation, comparing “brexit” and the second most discussed theme, “ukraine”.

**Figure 2:**
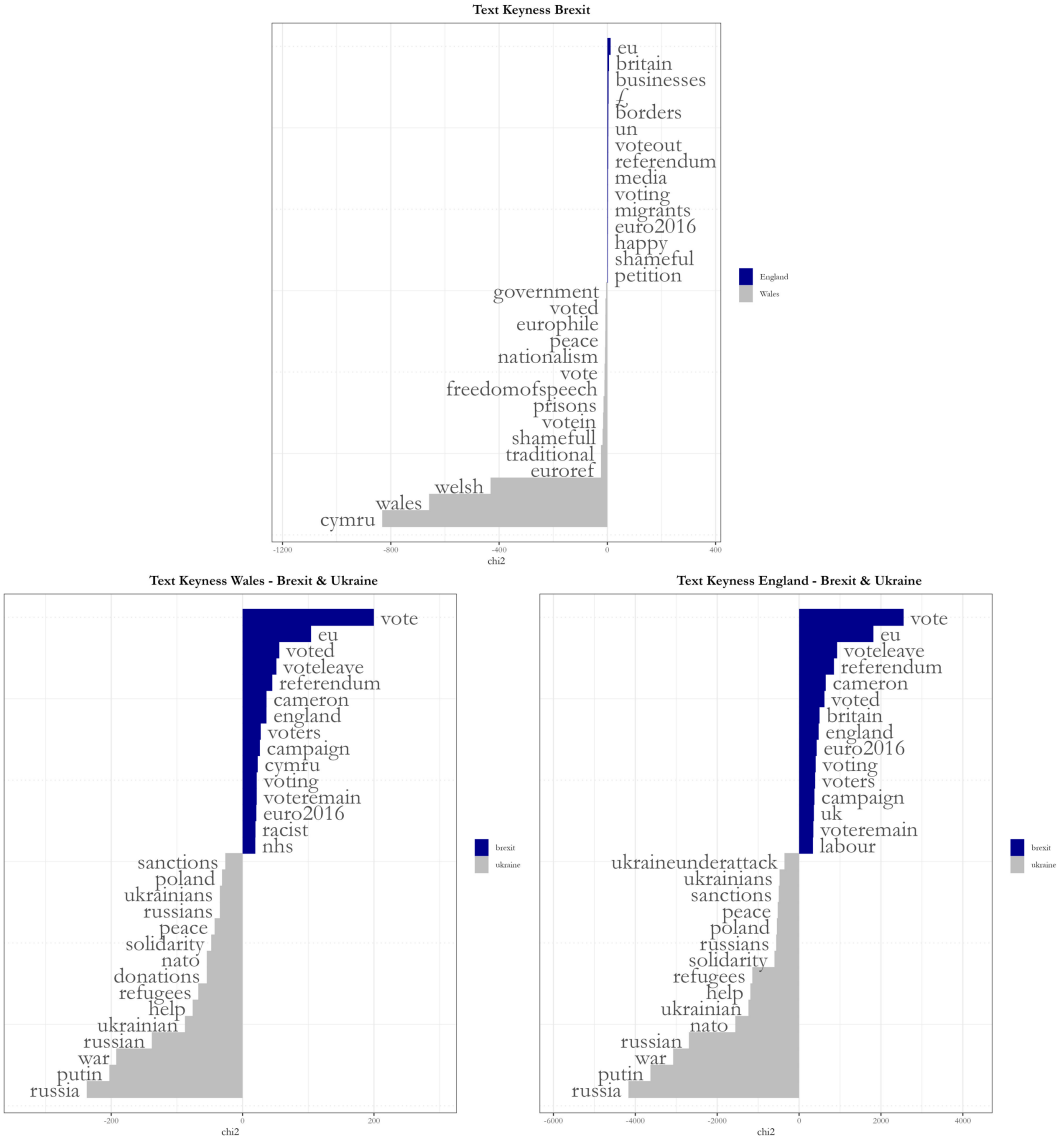
Keyness analysis, comparing Wales and England on Brexit and each nation on Brexit and Ukraine. Source: the author, with data from the Twitter API.

There are marked differences between each plot (Figure 2), as the size of the corpora did, in fact, affect the results. From the top plot, we can see that both the EU and Britain were more salient for English tweeters, with Welsh identity being a more salient topic for their Welsh counterparts. Notably, Cymru, the Welsh language name for Wales, had the top salience. Whilst “voteout” and “votein” appeared, respectively, for England and Wales, both also demonstrated conflicting words and, possibly, emotions around the topic, something we can also see in the bottom two charts, where “voteleave” and “voteremain” appear for both nations. Incidentally, for the other three themes, both nations were remarkably similar in discourse, demonstrating sentiments for the protection of refugees and, in the case presented, against the war on Ukraine.

**Figure 3:**
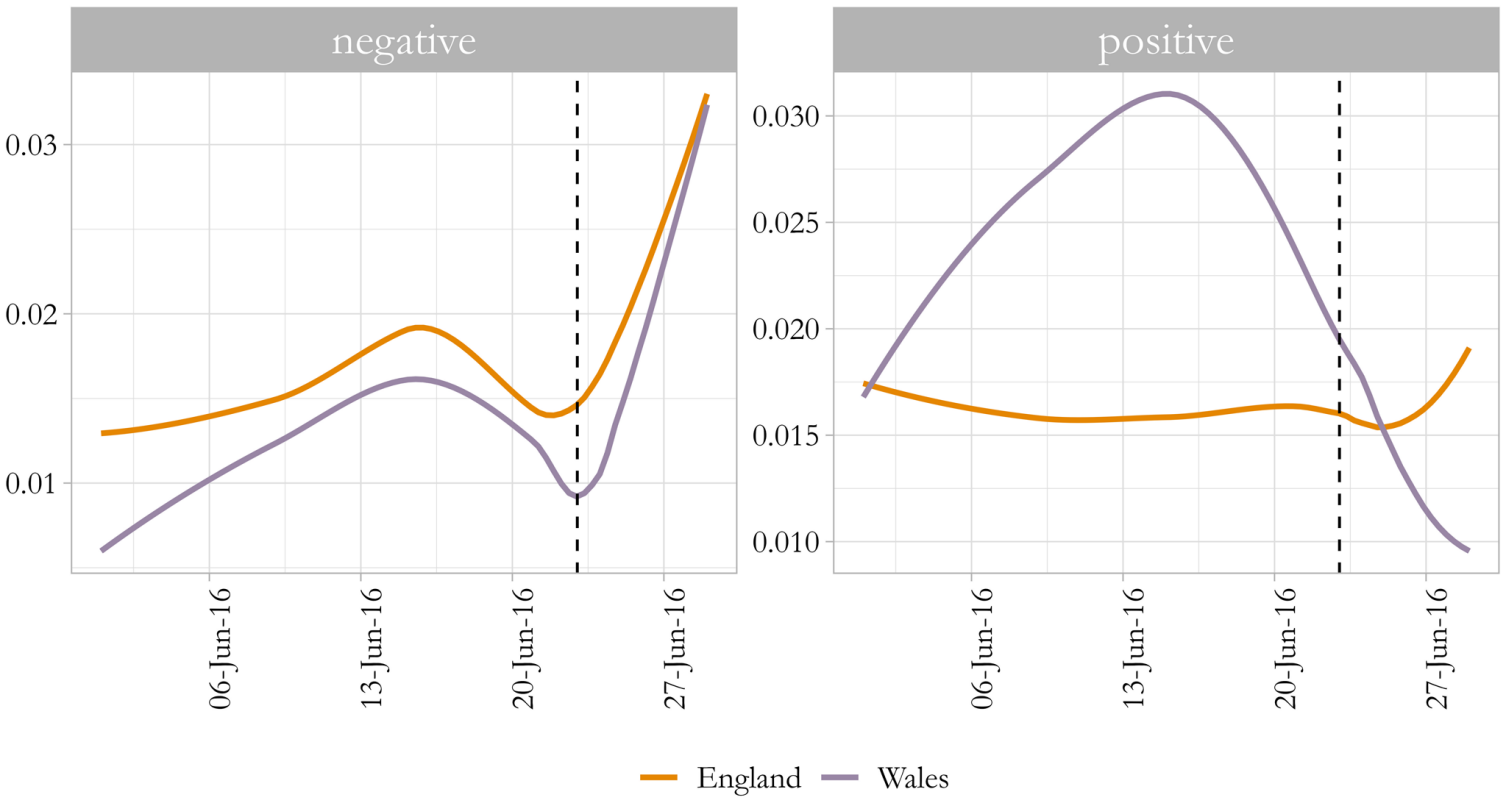
Sentiment analysis on tweets about Brexit over the month of June 2016, Wales and England. Source: the author, with data from the Twitter API.

A brief sentiment analysis of each nation’s corpus on Brexit shows that both nations turn particularly negative after the referendum and the positive sentiment present in Wales severely decreases afterwards. On the other hand, positive sentiment in England remained relatively constant throughout the month and slightly increased after 23 June. This is indicated by Figure 3, where negative and positive sentiment variation over time per nation is shown; the dashed line represents the day of the referendum, 23 June. Sentiment analysis of tweets, especially on controversial topics are difficult as they do not capture irony, sarcasm, or nuance.^7^ It does, however, enables us to have an overview image of the event.

One possibility for text-as-data analysis is topic modelling, using algorithms such as latent Dirichlet allocation (LDA) or structural topic modelling (stm) to identify what words are most likely to appear in a discourse or that are more relevant (Maier et al., 2018; Egger and Joanne, 2022). This is, again, a complex issue when it comes to short texts in relatively small corpora given that so much nuance is missed, but some results are discernible. To ensure that the size of the English corpus did not influence the Welsh topics, initial analyses were done separately.^8^

**Figure 4:**
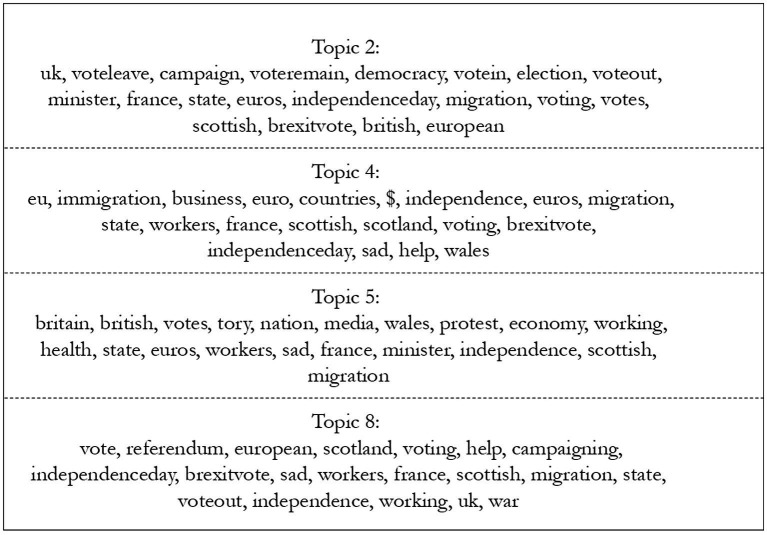
Most frequent and exclusive words in most salient topics for Wales, theme Brexit. Source: the author, with data from the Twitter API.

**Figure 5:**
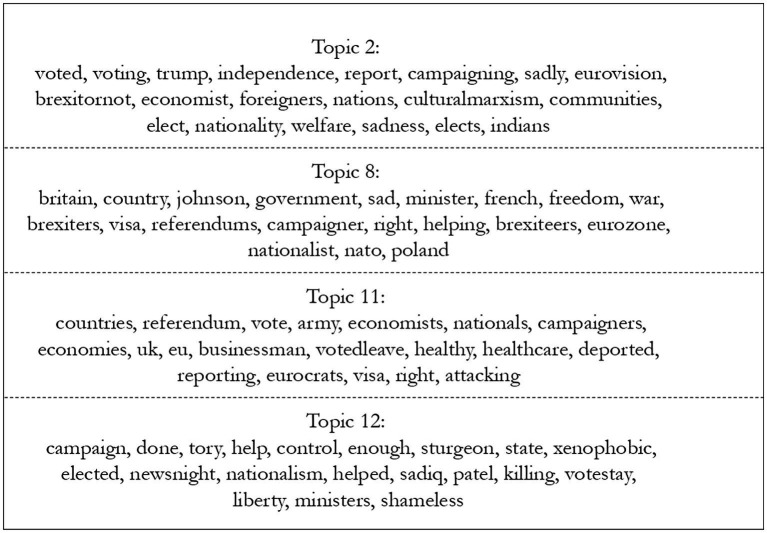
Most frequent and exclusive words in most salient topics for England, theme Brexit. Source: the author, with data from the Twitter API.

Figure 4 shows the most salient topics and their most frequent and exclusive words (FREX), that is, their most representative words, run with stm, for Wales. These seem to demonstrate a discourse that is amidst a debate, with conflicting opinions being included in different topics, rather than a massification of similar terms. Figure 5, showing FREX for England, indicates more consistency within topics, with the first three seemingly supportive of Brexit and the last, supportive of Remain. Although this is in line with expectations, it is possible that the difference in quantity of text leads to this variation in consistency.

Using the LDA algorithm to plot topics,^9^ we find that out of 2,734 observations of Welsh tweets, it assigns ten LDA topics, with a 10% probability, to 1,716 observations (62.8%). The most frequently assigned topic, with 193 observations, has the following most likely ten terms “country, voters, british, debate, campaign, britain, cameron, trump, immigrants, johnson.” The top ten tweets using the maximum *a posteriori* (MAP) estimation are related on Table 1. They demonstrate a disheartened perspective towards the results with a few strong pro-Brexit tweets.

**Table 1:** Top 10 tweets, from most frequent topic per nation, theme Brexit.

**Tweets**	**Nation**
Caretaker at work on merits of brexit “you will be fine, you work in security so crime will go up!’ Also “Boris makes me feel happy’	England
What a sad, sad, sad day to be British.I can totally believe the result, I knew it was coming. But it still awful. EURef Brexit	England
@eddieizzard thanks so much for all the brilliant work you have done to swing people to Brexit you and Bob Geldof have done a great job 4 us	England
What Britain has done is a massive step for all Europe. Other countries are now following suit. Took Britain to have the balls. Brexit	England
100 years ago Britain came in to a bloody war because of a treaty with Belgium 77 years later another war again won no respect Brexit	England
@jessicaelgot the Secretary of state for work is late! What is happening to British politics these days? Brexit LondonStays safetypin	England
Hate crime on the rise; economic disaster; and a gov in turmoil. What a wonderful country brexit EUref	England
The EU – a crime gang called communists – their op. is based around cultural marxism – Brexit INVOKEARTICLE50	England
This Britain is not my Britain. I want my country back BREXIT	England
Everyone who I have met in the lift, on the roads, at work, in the pub everyone is sad – who is happy then? Brexit unhappylondon	England
	
Boris Johnson for Prime Minister seems very likely. Will this result in the return of the country so wanted by the Brexit voters?	Wales
Make Britain Great again – Take our country back – We want our country back – Today is Independence Day – Brexit	Wales
@Nhawkins90 I would not worry about Russia mate! If this country vote Brexit no one can afford to go to Russia anyway 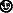 l’m in 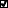 SOS 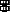	Wales
I want my country back from the hateful bastards that have been unleashed by Brexit vote. BritainAdrift	Wales
The leave campaign blatantly lied to voters who belueved them. Surely this cannot be legal? How should it be challenged. Remain Brexit	Wales
Brexit I thought Cameron and BorisJohnson both spoke well. Farage not so good. Triumphalism is not the British way.	Wales
The BREXIT Voters – taking the ORY out of COUNTRY 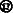 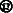 @RemaininEU	Wales
BREXIT voters – watch and listen very carefully. It has not taken long for the champions of your campaign to redact	Wales
Let improve our Great Britain and have a fresh start! brexit voteout LeaveCampaign	Wales
Brexit team on BBCDebate r putting up a better debate than Remain Remain r struggling 2 keep up with the debate with a petty comeback.	Wales

Source: the author. Tweets were unchanged apart from unreadable characters.

Source: the author, with data from the Twitter API.

Conducting the same analysis for English tweets, starting with 46,066 observations, we find that the model assigns topics to 28,534 (61.9%). The most frequently assigned topic, with 1,993 observations, has the following most likely ten terms “britain, done, country, work, sad, europe, job, nation, economic, british.” These show more varied views than the stm modelling before, with celebration, fear, and commiseration among them. In both cases, some irony and sarcasm leads us to consider the sentiment classification; however, there is no indication would be enough to drastically alter results.

**Figure 6:**
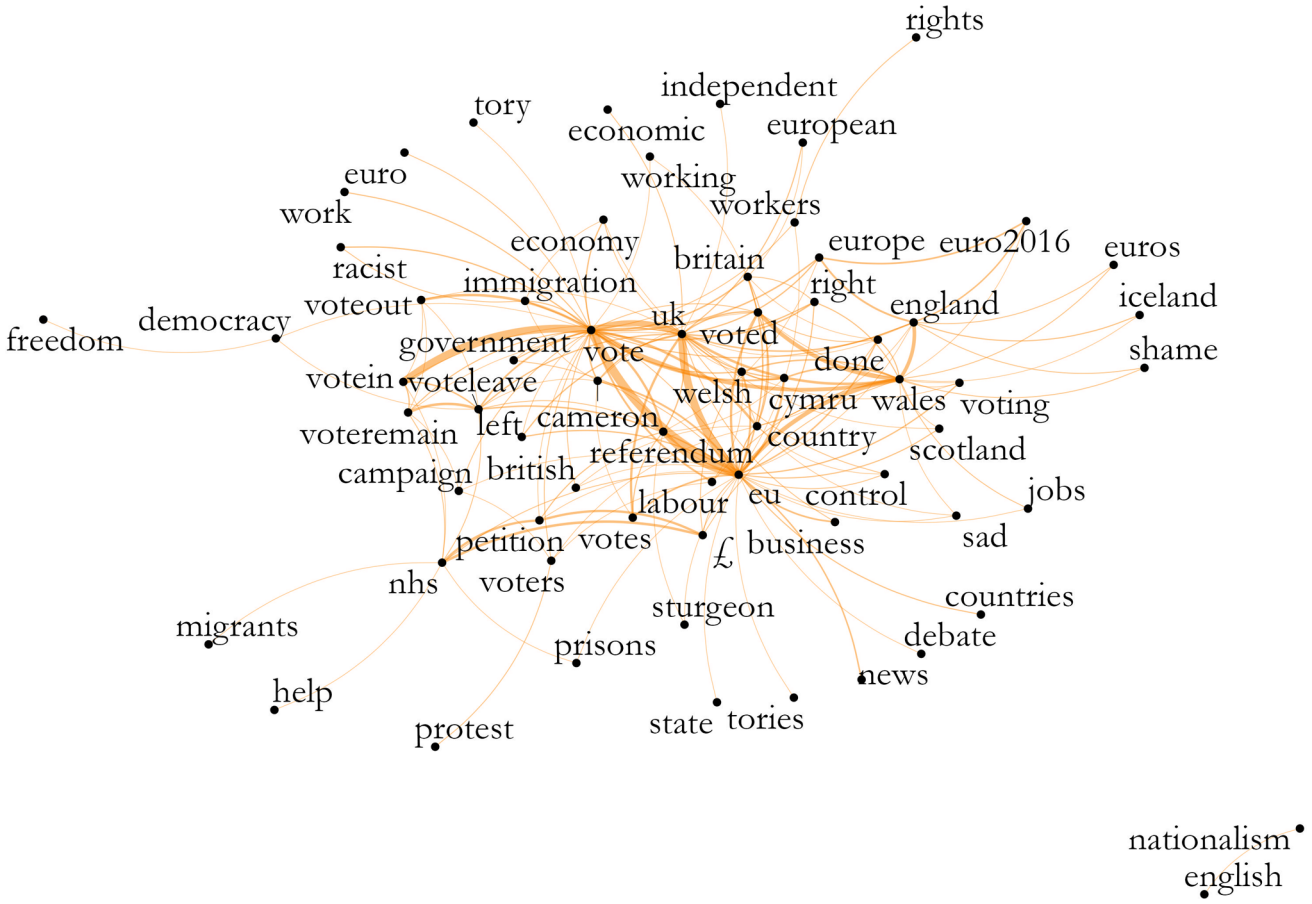
Co-occurrence of features, Wales, theme Brexit. Source: the author, with data from the Twitter API.

**Figure 7:**
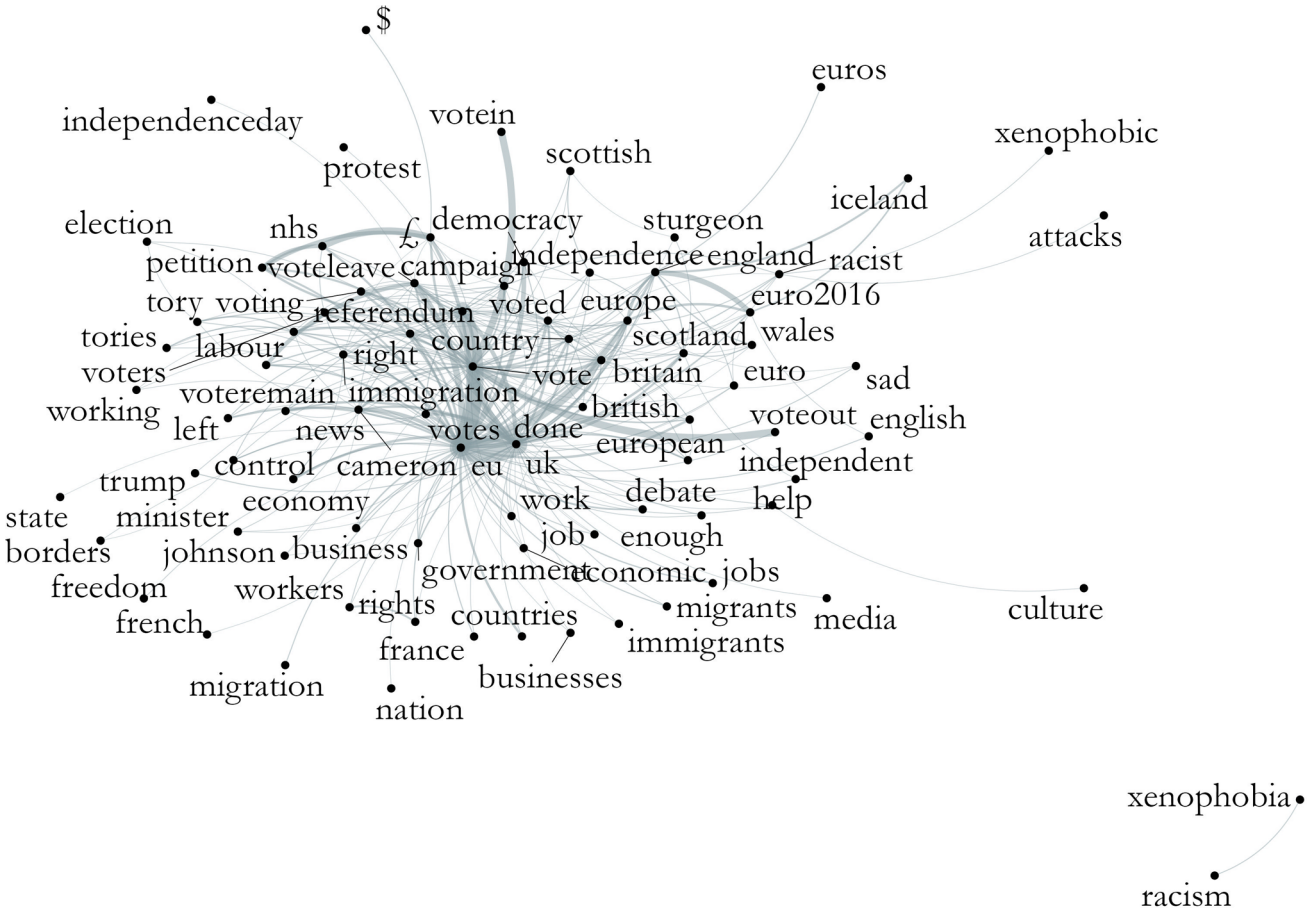
Co-occurrence of features, England, theme Brexit. Source: the author, with data from the Twitter API.

These are not highly specific results, as limits of space do not allow for all topics to be analyzed fully. Moreover, LDA and stm give slightly varying images given their different measurements. Using the co-occurrence of features, we have an image of which words or expressions (rather than topics) are used most often in connection with each other. Figures 6, 7 show these plots for Wales and England, respectively.^10^

We can identify the keyness results from above in the plot, now understanding better the connections among them. For Welsh tweets, the words that centered the discussion were “vote” and “referendum,” branching out to “government” and words related to Wales itself. We can also identify the relevance of economy and immigration-related words, although they are not always connected to each other; a relatively strong connection is present between “vote-immigration-voteout.”

For English tweets, the same words center the discussion, with “voteleave” strongly connected to “nhs” and a relatively strong connection between “left-voteremain-immigration,” although we cannot presume what “left” means in this context. Another strong connection can be identified between “vote-europe-independence-euro2016.” Both plots present outside nuclei with Wales showing “nationalism-english” and England “xenophobia-racism.” Both also show the connection between the NHS, the pound sign, and “petition”, referring to retweets with a petition for the “£350 million a week to the NHS’ promised by the Brexit campaign. These plots enable us to see a different type of overall discourse happening in these corpora.

**Figure 8:**
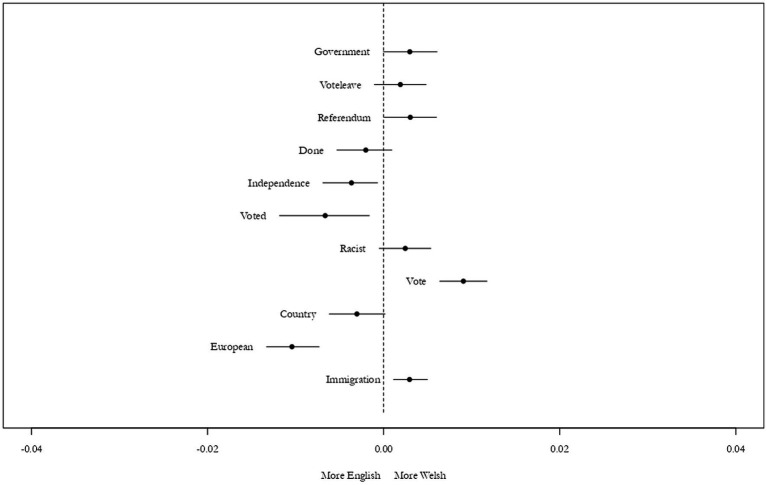
Topic association per nation, Brexit. Source: the author, with data from the Twitter API.

The identification of themes within topics can be quite tricky and requires care and transparency (Maier et al., 2018; Weston et al., 2023). In this case, words that are seemingly at odds often appear together (such as someone extorting leave voters to vote remain). Therefore, to maintain clarity, I have chosen to use the first word assigned as frequent and exclusive (FREX) to denominate a topic theme and have indicated all label topics in the Appendix. Figure 8 below shows, out the 15 topics previously identified as the most different, the 11 that most varied across English and Welsh tweets. For example, the topic “Voted,” included the words “euros2016, killed, working-class, helpful, ethnic, refugee”; meanwhile, the topic “Vote,” included “vote, euro, france, nation, depressing, economics, helps.” Despite the appearance that some of these seem more representative than others, other topics would have led to a possible argument of selective choosing, meaning a straightforward decision was the best outcome.

Looking at the tweets themselves, the content can vary widely from voting for “leave” to stop immigration to stating one’s ethnic minority status and a support for “leave”, to calling “leave” voters “racists”. There are 159 tweets that specifically use the word “regret” to refer to Brexit, including news reports, users who regretted their “leave” vote, and users calling out those who vote “leave” and were now regretting it. There were no tweets from “remain” supporters regretting their votes. The majority of high frequency tweeters (more than 50 tweets from the same user) come from England (51 to 3, a 5.6% proportion). On the other hand, tweeting fewer than five times was the norm for the majority of users, with 15,420 English users and 870 Welsh users tweeting at that frequency (5.6%). Therefore, we must take into account the possibility for outliers – high frequency tweeters shaping the corpora. Indeed, the user with 351 tweets was pro-Brexit; the Welsh tweeter with the most content tweeted 263 times and was also pro-Brexit. A cursory analysis of both high and low frequency tweeters showed similar results – while some of the features from high-frequency tweets do disappear when only low-frequency tweets are analyzed, the same level of contradiction and the same category of top used words appear for both nations.

A qualitative categorisation of tweets based on the topics extracted both quantitatively and through the author’s reading of the dataset indicated that words associated with politics, unsurprisingly, were the most frequent among both Welsh and English tweets, respectively, 36 and 21%. References to country or nationality came second, with 13% for both. This categorisation can be found in the Appendix.

Lastly, using a keyword-in-context check from the quanteda package, I was able to isolate all tweets referring to race, immigration, and ethnicity, as well as the main topic related to the feeling of “left-behind”, the NHS.^11^ Search for other public service related words did not turn up results, leading to the conclusion that indeed the NHS was the center of debate regarding Brexit and its alleged benefits.

There were 2,477 tweets from English tweeters and 135 from Welsh users (5.4%). There was a total of 712 tweets about the NHS, with 164 reposting or quoting the following “Now keep the promise of £ 350 m a week for our – Sign the petition: EuRef Leave Brexit via @38_degrees.” Some do add their own views, but the majority are simply a retweet, a “copy and paste” which means it is not possible to determine if they were originally supporters or against Brexit. Tweets that used the pound sign not often mentioned public services other than the NHS, but savings, deficits, and the economy. Only 100 tweets combine these with the keyword “*migrat*”. Within those, the seven Welsh tweets were claiming immigration to be an inherent problem, but some claiming that Brexit is a ploy to instigate privatisations or bring in cheap skilled labor.

There were 47 tweets from Welsh users about the race, immigration, and ethnicity and these are more consistent in claiming Brexit to be racist and wanting Remain as a view of “tolerance and unity”. This is not, however, the position taken by all tweets, simply a majority of them. There are 832 English tweets, and many focus on specific politicians such as Boris Johnson or David Cameron, as attack or defense, ask for promises to be kept, defend Brexit voters against claims of racism and anti-immigration sentiment; they also express solidarity to those who experienced racism following the referendum or defend the importance of immigration. On the other hand, there are also many who claim “free movement” and an excess of immigrants who “do not pay their share” is the root of the problem. Many perceive Brexit voters to have been lied to and led astray.

Although other research has focused on the quantity of data that Twitter can bring, a geotagged analysis mean less content and a focus on the geographical dynamics rather than the conversation itself. That is, given that geographical borders are somewhat reproduced on Twitter, once we hone in to one theme in one location, the differences in how Twitter actually functions for different areas can surface.

The Twitter metrics show that rather than a verified account having many replies and quote tweets, the Welsh pattern is to have single tweets without much interaction. Although higher engagement was expected from English Twitter, specifically with verified accounts generating many replies, this did not happen in the month of the referendum, despite the high traffic on the website. Quote tweets^12^ are used more often, as they are thought to generate more engagement to the user that quotes rather than the original tweeter, thus lowering the reply numbers.

## 5 Conclusion

Wales differs strongly from other British regions in history, language, population, type of government, and political ideologies. There is a dearth of regional research into Wales, and Welsh communities are indeed varied within the nation itself. Therefore, the analytical benefits from separating Wales from England should be self-evident and hopefully have been reinforced in this paper.

This paper has questioned whether English and Welsh communities on Twitter differed in their debate of Brexit and if that included discussions on race, immigration and a feeling of “left-behind”. It avoided direct comparisons given the difference in size for each corpus, aiming to analyze each nation on its own. Nevertheless, it found several issues were raised during the month of the referendum, and that these varied across England and Wales. Wales is a small nation in comparison to England and this is reflected in the amount of tweets collected. A methodological choice was made to confine the search to a timeframe, rather than a similar number of tweets. The frequency plots show how this is relevant to capture a topic on Twitter when it is being most talked about. While not representative of the Welsh and English populations respectively, the dataset has been shown to be representative of the community of Welsh and English tweeters.

Twitter is a self-selecting community of internet users. Internet access has increased widely across regions in both England and Wales, therefore, it can be argued that those who wish to be on Twitter are not stopped by lack of resources. Nevertheless, Twitter usage has never surpassed or even challenged that of Facebook. To use Twitter means to already be interested in posting your feelings, thoughts, and opinions to, in principle, strangers. The use of hashtags means attempting to insert oneself into a conversation and to mention someone, especially a verified member such as politician or media outlet, means possibly subjecting yourself to a wider audience. Therefore, the caveat that the population represented here is not statistically significant is essential. However, these are the people who chose to say something and reflect public opinion in real time.

The majority of tweets are not engaged with in any way, or gather maybe one or two “likes”. Given that the vast majority of tweets are not interacted with at all, there is something to be said about engagement and screaming into the void. However, by limiting my sample, I get a closer look at routine Twitter life, even if we consider these significant events that impact it. Welsh users seem more engaged with other users (who may or may not be Welsh), with higher percentages of replies and retweets overall, but they do not seem to receive the same level of response. Wales is a small community and its Twitter community smaller still. Although Twitter reproduces echo-chambers (Gorodnichenko et al., 2021), and does not necessarily link acquaintances as other networks do, it is possible that users know each other or would be able to identify each other, making Welsh twitter is possibly not as anonymous as one would expect. Another point to be made is who chooses to tweet and what about; it is no secret that social media algorithms thrive on conflict (Gorodnichenko et al., 2021; Larrauri et al., 2022; Stray et al., 2023) and some issues will drive individuals to tweet more and more extremely.

When focussing the analysis on the specific keywords of race, immigration, and ethnicity and the main driver of the “left-behind” feeling – the NHS – the tweets show that while these might have been tangentially connected, overall, users would concern themselves with one or the other. There are several other words that would lead to a discussion on migration, the central words comprise less than 5% of the full dataset. English tweets were more likely to take on aggressive stances and supporting Brexit due to immigration – with a few being only against EU immigration and believing there would be an easier access for other countries and regions to the UK and its market. Welsh tweeters mostly position themselves either for immigration, including due to its aging population, or sceptic that Brexit would actually curb it. Undoubtedly, however, there are those that were for Brexit and against immigration; as there were, in similar proportions, those in the English tweets commiserating the “racist undertones” of Brexit.

Overall, it would seem that being “left-behind” did in fact play a part in the discourse, but not necessarily as expected, as many doubted the NHS “£350 million a week” promised by the Brexit campaign and demanded the promise was kept. The issue of race and immigration was explored differently by each nation’s population, but not as strong as expected. The English tweets show a population that was much more conflicted about the topic, and the Welsh tweets do not show a population completely in agreement with the nation’s foundational myth.

## Data availability statement

Data and code available at: https://github.com/larissabr/tweetsdontvotepaper Further inquiries can be directed to the corresponding author.

## Author contributions

The author confirms being the sole contributor of this work and has approved it for publication.

## Footnotes

^1^Twitter has now been renamed X but was Twitter at the time of the events in question and the writing of this paper and therefore will be referred as such.

^2^As of 9 February 2023, the Twitter API has been paywalled, disrupting further data collection.

^3^The DPI, is a measure of household confidence in using government online resources. It was created as an alternative to the Hard-to-Count Digital Index.

^4^Throughout the paper, I have used “England” and “Wales” as synonymous to “English tweets” and “Welsh tweets”.

^5^A subsequent reproduction test collecting on multiple keywords over a one week period showed little difference in the results as far as how the conversation was centered around these topics during those times. Therefore, the one-month dataset was retained as the main focus of analysis.

^6^The topics were: Covid-19, Elon Musk, Eurovision, inflation, Liz Truss, the Oscars, Strictly Come Dancing, The Great British Bake Off, and the World Cup. They were distilled to main keywords if more than one was likely (i.e., ‘bakeoff’ and “british bake off”) and dates were chosen based on when they were relevant (i.e., during the airing of the season or Liz Truss’s premiership). The same process of supplementing the Twitter geotag was conducted, however using the place names already identified. The lowest ratio was during the Liz Truss government, with 97% English tweets and 3% Welsh tweets, and the highest ratio was for during the Strictly Come Dancing season, with 91 and 8%, respectively.

^7^The Lexicoder Young and Soroka (2012), which codes 4,579 negative and 4,569 positive words and expressions was chosen for this brief sentiment analysis of the Brexit corpora. Not only does this dictionary have the largest number of coded words, it also has a good balance between positive and negative, whereas many (such as bing, AFINN, and NRC) will have more negative words coded.

^8^To account for different sizes, the document-feature matrices were trimmed differently, requiring more frequency of terms and documents in the English dfm (seven and three, respectively, and checking for 15 topics) and fewer for the Welsh dfm (five and two, with 10 topics). The number of topics was tested previously.

^9^LDA uses Bayesian statistics, therefore a set.seed (12345) was used for replicability. It is important to highlight that not using set.seed or using a different set of numbers would lead to different results, meaning that while these are a sample of the tweets, they can be considered a random sample, rather than a representative one as the topics also vary. One aspect that does not change is how many topics are assigned.

^10^For Wales, the top 120 features were used, with a minimum occurring frequency of three. For England, the top 100 features were used, with a minimum occurring frequency of 20. This was done due to the difference in size of each corpus and its effect on the visualization of each plot.

^11^Words used were: “race*,” “*migrat*,” “ethni*,” “asylum*,” “refugee*,” “nhs,” “£,” “health*.”

^12^A “quote tweet” is the act of retweeting and adding a comment.
