# Supplementary material for: Tweets don’t vote – Twitter discourse from Wales and England during Brexit
Source: Front Sociol. 2023 Nov 16;8:1176732. doi: 10.3389/fsoc.2023.1176732 (PMC10690362; doi:10.3389/fsoc.2023.1176732)
Supplement: Supplementary file 1 [file Data_Sheet_1.docx]

# Appendix

## Qualitative keywords selected to trim tokens

“across england”, “across europe”, “afghan people”, “after brexit”, “against iceland”, “against russia”, “against slovakia”, “against wales”, “another country”, “anti immigration”, “anti-brexit protest”, “armed forces”, “asylum claims”, “asylum seeker”, “asylum seekers”, “attending schools”, “author joseph”, “avon canal”, “backs brexit”, “bad news”, “bad thing”, “bbc news”, “beautiful country”, “big ben”, “black country”, “boris johnson”, “both sides”, “brave people”, “breaker remain”, “breaking news”, “bred welsh”, “brexit brexit”, “brexit campaign”, “brexit campaigners”, “brexit debate”, “brexit vote”, “brexit voter”, “brexit voters”, “brighton england”, “britain votes”, “british airways”, “british army”, “british author”, “british boxing”, “british citizen”, “british citizens”, “british citizenship”, “british council”, “british electorate”, “british empire”, “british government”, “british history”, “british isles”, “british library”, “british museum”, “british passport”, “british people”, “british politics”, “british public”, “british red”, “british sports”, “british summer”, “british summertime”, “british troops”, “british tv”, “british values”, “british weather”, “british wrestlers”, “cameron resigns”, “cardiff bay”, “cash votes”, “champions league”, “channel tunnel”, “chick cocky”, “church of england”, “civil war”, “claim asylum”, “climate change”, “cmon england”, “c'mon england”, “cmon wales”, “c'mon wales”, “concentration camps”, “congestion delays”, “congestion moderate”, “conservative party”, “control immigration”, “control over”, “controlled immigration”, “country back”, “country club”, “country house”, “country mile”, “country music”, “country needs”, “country park”, “country retreat”, “country's future”, “crisis appeal”, “cross bred”, “cup final”, “customer service”, “cymru am byth”, “daily mail”, “dark day”, “dark fantasy”, “david beckham”, “david cameron”, “david cameron's”, “davids day”, “david's day”, “delay m4”, “delays possible”, “demand uk”, “democratic country”, “dewi hapus”, “didn't know”, “didn't realise”, “didn't think”, “didn't vote”, “didn't want”, “difference between”, “different country”, “diligence to”, “dirty russian”, “divided nation”, “doc ukraine”, “doesn't even”, “doesn't matter”, “doesn't mean”, “doesn't want”, “doing well”, “donald trump”, “done wales”, “downing street”, “drink outlet”, “dual nationality”, “due diligence”, “dydd gwyl”, “dydd gŵyl”, “easter sunday”, “economic migrants”, “eddie jones”, “el brexit”, “end up”, “ended up”, “england captain”, “england fan”, “england fans”, “england game”, “england manager”, “england match”, “england play”, “england rugby”, “england shirt”, “england squad”, “england u.k”, “england uk”, “england's best”, “english breakfast”, “english channel”, “english football”, “english language”, “english lit”, “english literature”, “english players”, “english speaking”, “english summer”, “english teacher”, “english teams”, “english version”, “english weather”, “entire country”, “especially when”, “et al”, “ethnic minorities”, “ethnic minority”, “eu cash”, “eu citizens”, “eu countries”, “eu does”, “eu funding”, “eu membership”, “eu migration”, “eu referendum”, “european countries”, “european country”, “european union”, “eurovision song”, “even worse”, “ever seen”, “every day”, “every game”, “every other”, “every single”, “every time”, “everyone else”, “everyone who”, “exactly what”, “expected to”, “fact that”, “fair play”, “fake news”, “fake revolt”, “falling apart”, “fantasy horror”, “far away”, “far more”, “far right”, “far too”, “feature shout”, “fed up”, “feel ashamed”, “feel like”, “feel sick”, “feel sorry”, “feels like”, “felt so”, “few days”, “few hours”, “few weeks”, “few years”, “find out”, “fingers crossed”, “finish top”, “finishing second”, “fintan o'toole”, “first day”, “first half”, “first place”, “first safe”, “first time”, “flag profile”, “fleeing from”, “fleeing ukraine”, “fleeing war”, “fly zone”, “follow the”, “following brexit”, “food poisoning”, “football fans”, “football team”, “for africans”, “for christmas”, “for decades”, “for example”, “for peace”, “for processing”, “for sale”, “for tickets”, “for years”, “forced to”, “foreign policy”, “forget about”, “forward to”, “found out”, “fourth book”, “free movement”, “free preview”, “friday shout”, “frinton-on-sea england”, “from france”, “from london”, “from outside”, “from runcorn”, “ftt resets”, “fuck off”, “fuck sake”, “fucked up”, “full english”, “funny how”, “future generations”, “game tonight”, “gareth bale”, “general election”, “genuine refugees”, “george osborne”, “george soros”, “germany france”, “get away”, “get here”, “get knocked”, “get rid”, “get through”, “give him”, “give me”, “give them”, “give up”, “give us”, “glad i”, “glory to”, “go back”, “go down”, “go home”, “go through”, “god bless”, “god help”, “going through”, “going to”, “good enough”, “good friday”, “good idea”, “good job”, “good luck”, “good morning”, “good news”, “good old”, “good reason”, “good thing”, “got money”, “government must”, “granted asylum”, “great again”, “great britain”, “great british”, “great idea”, “great result”, “great start”, “great uncle”, “grew up”, “group b”, “grow up”, “gt gt”, “gwyl dewi”, “gŵyl dewi”, “ha ha”, “had enough”, “half time”, “happened to”, “happens next”, “happens now”, “happens when”, “happy birthday”, “happy easter”, “happy independence”, “happy saint”, “happy st”, “hapus happy”, “has anyone”, “has changed”, “has decided”, “has done”, “has fallen”, “has given”, “has gone”, “has happened”, “has lost”, “has made”, “has resigned”, “has spoken”, “has taken”, “has won”, “have already”, “have any”, “have changed”, “have decided”, “have destroyed”, “have donated”, “have done”, “have gone”, “have seen”, “have spoken”, “have taken”, “have tried”, “have turned”, “have watched”, “have woken”, “haven't got”, “haven't seen”, “having a”, “he did”, “he didn't”, “he does”, “he doesn't”, “he gets”, “he has”, “he lied”, “he needs”, “he plays”, “he said”, “he says”, “he should”, “he wants”, “he was”, “he won't”, “help provide”, “help ukraine”, “help us”, “here's my”, “he's doing”, “hey twitter”, “higher than”, “hijacked to”, “him how”, “his family”, “his mp's”, “his own”, “home immediately”, “home nation”, “home nations”, “home office”, “home secretary”, “hope you”, “hope you're”, “hoping this”, “horror story”, “how bad”, “how can”, “how dare”, “how did”, “how does”, “how far”, “how long”, “how many”, “how much”, “huge anti-brexit”, “human rights”, “humanitarian appeal”, “illegal immigrant”, “illegal immigrants”, “illegal immigration”, “illegal migrants”, “i'm voting”, “immigration policy”, “immigration system”, “independence day”, “independent country”, “independent nation”, “innocent civilians”, “innocent people”, “interest rates”, “international law”, “invaded ukraine”, “invading ukraine”, “invasion of ukraine”, “irish passport”, “jeremy corbyn”, “jo cox”, “joe allen”, “joe root”, “john oliver”, “johnson gove”, “join me”, “join us”, “jordan henderson”, “joseph roy”, “keep out”, “kicked out”, “labour party”, “labour voters”, “lake district”, “last year”, “last years”, “law breaker”, “law breaking”, “le pen”, “leave because”, “leave campaign”, “leave campaigners”, “leave voters”, “leeds england”, “legal routes”, “legally binding”, “lib dems”, “little england”, “little englanders”, “local communities”, “local elections”, “london england”, “london united”, “long term”, “long time”, “look after”, “look like”, “looking good”, “looks like”, “lunatics have”, “made me”, “major tournament”, “make britain great again”, “mass immigration”, “mass migration”, “mental health”, “michael gove”, “middle england”, “miles away”, “million people”, “moderate delay”, “mp's back”, “mps demand”, “n ireland”, “narrow minded”, “nation divided”, “nation state”, “national anthem”, “near hungerford”, “new pm”, “new zealand”, “newport wales”, “next door”, “next election”, “next few”, “next game”, “next general”, “next pm”, “next round”, “next time”, “next week”, “next year”, “next years”, “nicola sturgeon”, “nigel farage”, “no chance”, “no confidence”, “no doubt”, “no fly”, “no idea”, “no longer”, “no matter”, “no plan”, “no surprise”, “no way”, “no wonder”, “no words”, “no-fly zone”, “non eu”, “north east”, “north wales”, “north west”, “northern ireland”, “northern irish”, “not allowed”, “not even”, “not illegal”, “not sure”, “not surprised”, “nothing else”, “now clear”, “now keep”, “now let's”, “nuclear power”, “nuclear war”, “nuclear weapons”, “offshore camps”, “old cross”, “old people”, “older generation”, “ordinary people”, “other countries”, “other half”, “other mp's”, “other news”, “other side”, “other than”, “other way”, “our borders”, “our children”, “our country”, “our country's”, “our economy”, “our future”, “our lives”, “our own”, “our politicians”, “our sign”, “our wee”, “out dirty”, “over half”, “over here”, “over years”, “own country”, “own flag”, “own goal”, “park road”, “partygate this”, “paul mason”, “pay rise”, “people fleeing”, “people saying”, “people smugglers”, “people traffickers”, “people who”, “performance from”, “person who”, “personally i”, “petition via”, “pic what”, “pick up”, “picked up”, “piss poor”, “plan b”, “plan immediately”, “planning to”, “plans to”, “play iceland”, “played well”, “playing well”, “please checkout”, “please donate”, “please don't”, “please help”, “please share”, “please sign”, “please stop”, “please support”, “please tell”, “pleasure to”, “plus side”, “pm how”, “poland wales”, “political party”, “polling station”, “polling stations”, “port talbot”, “possesion shots”, “post brexit”, “premier league”, “pretty much”, “pretty sure”, “preview buy”, “prime minister”, “prince of wales”, “priti patel”, “private schools”, “pro brexit”, “profile pic”, “project fear”, “protest at”, “proud to”, “provide aid”, “provide safe”, “public accept”, “public services”, “pull together”, “put up”, “putin apologist”, “quarter final”, “quarter finals”, “raise money”, “re brexit”, “reading england”, “red cross”, “redditch england”, “reduce immigration”, “referendum result”, “referendum rules”, “referring to”, “refugee crisis”, “refugees arriving”, “refugees fleeing”, “refugees from”, “refuse to”, “refused to”, “refuses to”, “refusing to”, “remain as”, “remain camp”, “remain campaign”, “remain voters”, “remember when”, “reminder that”, “reminds me”, “resets uk”, “revolt working-class”, “right wing”, “rights record”, “robbie savage”, “roman abramovich”, “roy hodgson”, “roy wright”, “rugby team”, “rules triggering”, “russian army”, “russian children”, “russian forces”, “russian invasion”, “russian military”, “russian money”, “russian oligarchs”, “russian soldiers”, “russian troops”, “russia's invasion”, “rwanda deal”, “rwanda plan”, “rwanda policy”, “rwanda scheme”, “sad day”, “sad sad”, “sadiq khan”, “safe country”, “safe passage”, “safe routes”, “said he”, “saint david's”, “same old”, “same thing”, “same time”, “same way”, “saturday night”, “save our”, “says he”, “says it”, “schools ramp”, “schools uk”, “scottish independence”, “second eu referendum”, “second half”, “second referendum”, “seek asylum”, “seeking asylum”, “self serving”, “semi final”, “send asylum”, “send them”, “sending asylum”, “sending people”, “sending refugees”, “sent home”, “set up”, “severe delay”, “severity moderate”, “shadow cabinet”, “shane warne”, “she said”, “she was”, “sheffield england”, “short term”, “single market”, “sky news”, “slava ukraini”, “small minded”, “social media”, “song contest”, “south africa”, “south east”, “south wales”, “southampton england”, “sovereign nation”, “speak english”, “sports person”, “sri lanka”, “st davids”, “st david's”, “stay eu”, “still undecided”, “stop immigration”, “sturgeon says”, “suffering unbelievably”, “suggestions that”, “je suis european”, “survive partygate”, “take back control”, “take control”, “take over”, “taken our”, “taken over”, “this country”, “those fleeing”, “tom daley”, “tomorrow night”, “tony blair”, “tory government”, “tory mps”, “tory party”, “totally agree”, “trade deal”, “trade deals”, “triple lifesaver”, “truly fucked”, “trust me”, “typical england”, “uk economy”, “uk government”, “uk private”, “uk should”, “uk votes”, “ukraine crisis”, “ukraine flag”, “ukraine humanitarian”, “ukraine invasion”, “ukraine refugees”, “ukraine sign”, “ukraine suffering”, “ukrainian people”, “ukrainian refugee”, “ukrainian refugees”, “ukranian refugees”, “uncontrolled immigration”, “undead rise”, “unimaginable cruelty”, “united kingdom”, “vladimir putin”, “vote leave”, “vote remain”, “voted for”, “voted leave”, “voted out”, “voted remain”, “votes leave”, “voting leave”, “voting out”, “voting remain”, “wales game”, “wales match”, “wales northern”, “wales town”, “wales won”, “want to”, “war crime”, “war crimes”, “war criminal”, “warrington england”, “watching england”, “wee country”, “welsh assembly”, “welsh business”, “welsh businesses”, “welsh cake”, “welsh cakes”, “welsh cob”, “welsh economy”, “welsh fans”, “welsh fire”, “welsh flag”, “welsh football”, “welsh friends”, “welsh government”, “welsh independence”, “welsh language”, “welsh national”, “welsh open”, “welsh rugby”, “west country”, “west wales”, “work together”, “workers rights”, “working class”, “working-class culture”, “world class”, “world cup”, “world war”, “worried about”, “worry about”, “worse off”, “worse than”, “worst thing”, “york england”, “young men”, “young people”, “your mp”, “crossing the channel”, “small boats”, “channel crossing”, “channel crossings”, “helmand province”, “dominic raab”, “proudly serving”, “queen and country”, “june 23rd”, “23rd june”, “better in” “immigra*”,”migra*”, “asylum*”, “refugee*”, “xenophob*”, “foreign*”, “racis*”, “ethnic*”, “muslim*”, “hindu*”, “indian*”, ““, “origin*”, “country of origin”, “turban*”, “towel*”,

“tunic*”, “hijab*”, “veil*”, “niqab*”, “head cover”, “religion”, “tradition*”,

“white”, “black”, “brown”, “paki”, “asian”, “country”, “countries”, “nation*”, “identit*”, “cultur*”, “slovakia”, “iceland”, “poland”, “polish”, “ukrain*”, “rwanda*”, “pakistan*”, “afghan*”, “france”, “french”, “england”, “english”, “wales”, “welsh”, “british”, “britain”, “uk”, “united kingdom”,

“cymru”, “byth”, “scotland”, “scottish”, “united states”, “united nations”, “un”, “eastern”, “middle east*”, “estate*”, “nato”, “north atlantic”, “council*”, “putin”,”oligarch*”, “donation*”, “communit*”, “social hous*”, “social care*”, “welfare*”, “business*”, “nhs”, “national health service”, “health*”, “job*”, “sanction*”, “condemn*”, “help*”, “solidarity”, “solidary”, “£”, “$”, “€”, “eu”, “euro*”, “media*”, “news*”, “report*”, “elect*”, “campaign*”, “brexit*”, “work*”, “burden*”, “cameron”, “expat*”, “visa”, “crossing*”, “small boat*”, “channel cross*”, “channel*”, “deport*”, “send back”, “return”, “kill*”, “boat*”, “vot*”, “right*”, “right wing”, “left*”,”left wing”, “petition*”, “government*”, “peace*”, “attack*”, “unjust”, “unfounded”, “war”, “crim*”, “democrac*”, “army”, “armed forces”, “local authorit*”, “border*”, “control*”, “referendum*”, “independen*”, “prison*”, “debate*”, “public service*”, “devolution”, “devolved”, “econom*”, “russia*”, “sturgeon”, “public fund*”, “avon”, “trump”, “biden”, “johnson”, “patel”, “drakeford”, “labour”, “tory”, “tories”, “minister*”, “state”, “partygate”, “freedom*”, “libert*”, “autonom*”,”self-determination”, “determination*”, “shame*”, “happy”, “sad*”, “depress*”, “protest*”, “enough”, “done”, “tired”, “reset*”

## Qualitative categorisation of keywords into topics

| Afghanistan | Brexit | Britain | Country/  nationality | Economy | England | Feeling | Identity/religion |
| --- | --- | --- | --- | --- | --- | --- | --- |
| afghan* | brexit* | britain | eastern | business* | england | depress* | communit* |
|  | euro* | uk | eu | econom* | english | donation* | cultur* |
|  | referendum* | british | europe* | job* |  | happy | hijab* |
|  |  |  | france | work* |  | petition* | hindu* |
|  |  |  | paki* | austerity |  | sad* | identit* |
|  |  |  | poland |  |  | shame* | muslim* |
|  |  |  | polish |  |  |  | nation* |
|  |  |  | russia* |  |  |  | tradition* |
|  |  |  | scotland |  |  |  | veil* |
|  |  |  | scottish |  |  |  | white |
|  |  |  | slovakia* |  |  |  |  |
|  |  |  | spain |  |  |  |  |
|  |  |  | spanish |  |  |  |  |

| Immigration/race | NHS | Person | Politics | Rwanda | Self-determination | Ukraine | Wales |
| --- | --- | --- | --- | --- | --- | --- | --- |
| asylum* | health* | cameron | army | rwanda* | autonom* | ukrain* | cymru |
| migra* | nhs | corbyn | council* |  | control* |  | byth |
| origin* |  | johnson | country |  | determination* | | wales |
| racis* |  | patel | crim* |  | devol* |  | welsh |
| turban* |  | putin | debate* |  | freedom* |  |  |
| visa |  | sturgeon | democrac* |  | libert* |  |  |
| avon* |  | drakeford | estate* |  |  |  |  |
| boat* |  | carywn jones | goverment* |  |  |  |  |
| border* |  | carywnjones | hous* |  |  |  |  |
| burden* |  |  | labour* |  |  |  |  |
| channel* |  |  | left* |  |  |  |  |
| crossing* |  |  | minister* |  |  |  |  |
| ethnic* |  |  | oligarchs |  |  |  |  |
| expat* |  |  | partygate |  |  |  |  |
| foreign* |  |  | prison* |  |  |  |  |
| immigra* |  |  | religion |  |  |  |  |
| refugee* |  |  | right* |  |  |  |  |
| towel* |  |  | sanction* |  |  |  |  |
| xenophob* |  |  | state |  |  |  |  |
|  |  |  | tories |  |  |  |  |
|  |  |  | tory |  |  |  |  |
|  |  |  | un |  |  |  |  |
|  |  |  | vot* |  |  |  |  |
|  |  |  | war |  |  |  |  |
|  |  |  | welfare* |  |  |  |  |

## STM Topics top words

*England*

Topic 1 Top Words:

Highest Prob: british, debate, work, economic, jobs, votein, protest

FREX: british, debate, work, economic, jobs, votein, protest

Lift: migrant, british, europeans, jobs, migration, businesses, crimes

Score: votein, british, debate, work, economic, jobs, migration

Topic 2 Top Words:

Highest Prob: voted, voting, trump, independence, report, campaigning, sadly

FREX: voted, voting, trump, independence, report, campaigning, sadly

Lift: elect, eurovision, trump, communities, culturalmarxism, elects, foreigners

Score: voted, voting, trump, independence, eurovision, report, sadly

Topic 3 Top Words:

Highest Prob: voteleave, labour, media, happy, reports, newspaper, europeanunion

FREX: voteleave, labour, media, happy, reports, newspaper, europeanunion

Lift: asylum, europeanunion, identity, killer, newspaper, happy, labour

Score: voteleave, labour, media, happy, channels, reports, newspaper

Topic 4 Top Words:

Highest Prob: cameron, business, white, attacks, return, brown, worked

FREX: cameron, business, white, attacks, return, brown, worked

Lift: attacks, council, crime, electoral, euromillions, foreigner, governments

Score: cameron, business, white, attacks, return, brown, europhiles

Topic 5 Top Words:

Highest Prob: voteremain, votes, rights, workers, shame, independenceday, putin

FREX: voteremain, votes, rights, workers, shame, independenceday, putin

Lift: attacked, economically, immigrant, independenceday, workers, rights, shame

Score: voteremain, votes, rights, workers, shame, independenceday, putin

Topic 6 Top Words:

Highest Prob: england, euro2016, european, iceland, wales, racists, electorate

FREX: england, euro2016, european, iceland, wales, racists, electorate

Lift: euro2016, helpful, slovakia, voteleavetakecontrol, iceland, wales, brexitin5words

Score: england, euro2016, european, iceland, wales, working-class, racists

Topic 7 Top Words:

Highest Prob: europe, democracy, voteout, english, tories, working, national

FREX: europe, democracy, voteout, english, tories, working, national

Lift: campaigned, depression, muslim, saddest, tired, attack, english

Score: europe, democracy, voteout, english, tories, working, national

Topic 8 Top Words:

Highest Prob: britain, country, right, johnson, government, sad, minister

FREX: britain, country, johnson, government, sad, minister, french

Lift: eurotrash, nationalist, refugee, french, government, johnson, right

Score: britain, country, right, sad, johnson, government, minister

Topic 9 Top Words:

Highest Prob: £, nhs, euro, election, france, immigrants, euros

FREX: £, nhs, euro, election, france, immigrants, euros

Lift: £, depressing, euro, euros, foreign, immigrants, original

Score: £, nhs, euro, election, euros, france, immigrants

Topic 10 Top Words:

Highest Prob: news, economy, racism, scottish, borders, border, reporting

FREX: news, economy, racism, scottish, borders, border, works

Lift: border, borders, economics, economy, news, newspapers, petitions

Score: news, economy, racism, newspapers, scottish, border, borders

Topic 11 Top Words:

Highest Prob: eu, vote, uk, referendum, countries, campaigners, economists

FREX: countries, referendum, vote, army, economists, nationals, campaigners

Lift: army, businessman, countries, nationals, referendum, economies, vote

Score: vote, eu, referendum, uk, countries, businessman, campaigners

Topic 12 Top Words:

Highest Prob: campaign, done, tory, help, control, enough, sturgeon

FREX: campaign, done, tory, help, control, enough, sturgeon

Lift: campaign, done, helped, nationalism, patel, sadiq, sturgeon

Score: campaign, done, tory, help, control, enough, sturgeon

Topic 13 Top Words:

Highest Prob: job, nation, migrants, independent, un, channel, euros2016

FREX: job, nation, migrants, independent, un, channel, euros2016

Lift: euros2016, nation, boats, brexitinfivewords, condemn, crossing, depressed

Score: job, nation, independent, migrants, un, channel, crossing

Topic 14 Top Words:

Highest Prob: voters, immigration, petition, racist, $, €, community

FREX: voters, immigration, racist, $, €, community, eastern

Lift: €, community, controlling, criminal, eastern, ethnic, europe's

Score: $, voters, petition, immigration, racist, €, community

Topic 15 Top Words:

Highest Prob: scotland, left, eu, asian, helps, deport, protesting

FREX: scotland, left, asian, helps, deport, protesting, eu

Lift: protesting, deport, eurostar, left, scotland, asian, helps

Score: scotland, left, eurostar, eu, asian, deport, helps

*Wales*

Topic 1 Top Words:

Highest Prob: welsh, news, job, johnson, white, sad, voting

FREX: welsh, news, job, johnson, white, sad, scottish

Lift: job, news, white, johnson, welsh, sad, scottish

Score: welsh, white, news, job, johnson, sad, voting

Topic 2 Top Words:

Highest Prob: uk, voteleave, campaign, voteremain, democracy, votein, minister

FREX: uk, voteleave, campaign, voteremain, democracy, votein, election

Lift: voteremain, campaign, uk, democracy, election, votein, voteout

Score: votein, uk, voteleave, campaign, voteremain, democracy, voteout

Topic 3 Top Words:

Highest Prob: country, cymru, racist, jobs, control, racism, immigrants

FREX: country, cymru, racist, jobs, control, racism, immigrants

Lift: control, jobs, racism, country, immigrants, voter, cymru

Score: country, cymru, voter, racist, racism, jobs, control

Topic 4 Top Words:

Highest Prob: eu, immigration, business, euro, independence, euros, countries

FREX: eu, immigration, business, euro, countries, $, independence

Lift: business, euro, $, countries, eu, immigration, independence

Score: eu, $, immigration, business, euro, independence, countries

Topic 5 Top Words:

Highest Prob: wales, britain, british, votes, economy, tory, nation

FREX: britain, british, votes, tory, nation, media, wales

Lift: protest, tory, votes, britain, british, health, media

Score: wales, protest, britain, british, economy, votes, tory

Topic 6 Top Words:

Highest Prob: europe, done, labour, euro2016, sturgeon, french, nations

FREX: europe, done, labour, euro2016, sturgeon, french, nations

Lift: french, labour, nations, sturgeon, done, euro2016, europe

Score: europe, french, done, euro2016, labour, sturgeon, nations

Topic 7 Top Words:

Highest Prob: voted, england, voters, government, rights, iceland, peace

FREX: voted, england, voters, government, rights, iceland, peace

Lift: shame, voters, england, rights, government, happy, iceland

Score: voted, england, shame, voters, government, rights, iceland

Topic 8 Top Words:

Highest Prob: vote, referendum, voting, european, help, scotland, sad

FREX: vote, referendum, european, scotland, voting, help, campaigning

Lift: referendum, campaigning, european, scotland, vote, brexitvote, independenceday

Score: vote, campaigning, referendum, european, voting, help, scotland

Topic 9 Top Words:

Highest Prob: cameron, right, nhs, £, left, debate, petition

FREX: cameron, right, nhs, £, left, debate, petition

Lift: debate, nhs, war, £, freedom, left, national

Score: nationalism, cameron, £, right, nhs, left, petition

Topic 10 Top Words:

Highest Prob: work, trump, enough, economic, english, campaigners, independent

FREX: work, trump, enough, economic, english, campaigners, independent

Lift: trump, english, enough, independent, work, campaigners, economic

Score: english, work, trump, enough, economic, campaigners, independent

*Brexit corpus (Wales and England)*

Topic 1 Top Words:

Highest Prob: uk, cameron, voters, left, votein, freedom, health

FREX: votein, voters, freedom, left, uk, europe's, cameron

Lift: solidarity, votein, voters, cameron, europa, europe's, freedom

Score: uk, votein, cameron, voters, left, freedom, health

Topic 2 Top Words:

Highest Prob: eu, europe, government, help, media, immigrants, cymru

FREX: government, cymru, help, referendums, eu, eurozone, europe

Lift: cymru, elects, eurozone, government, help, indians, nato

Score: eu, europe, government, help, media, immigrants, elects

Topic 3 Top Words:

Highest Prob: voteleave, labour, british, happy, independent, national, shame

FREX: voteleave, labour, british, happy, independent, national, shame

Lift: national, shame, voteleavetakecontrol, british, independent, labour, brown

Score: voteleave, labour, british, happy, shame, national, independent

Topic 4 Top Words:

Highest Prob: referendum, sad, tories, jobs, campaigners, white, welsh

FREX: referendum, sad, tories, jobs, campaigners, white, welsh

Lift: identity, sad, white, attacking, campaigners, council, europhiles

Score: referendum, sad, jobs, tories, campaigners, white, welsh

Topic 5 Top Words:

Highest Prob: done, debate, control, voteout, brexitvote, campaigning, black

FREX: done, debate, control, voteout, brexitvote, campaigning, black

Lift: electoral, lefties, nationalist, brexitvote, control, attacked, black

Score: done, debate, voteout, control, brexitvote, campaigning, black

Topic 6 Top Words:

Highest Prob: independence, sturgeon, scottish, migrants, racists, migration, polish

FREX: independence, sturgeon, scottish, migrants, racists, migration, polish

Lift: brexits, community, migrants, migration, polish, scottish, sturgeon

Score: independence, scottish, sturgeon, migrants, migration, racists, putin

Topic 7 Top Words:

Highest Prob: voted, euros2016, killed, working-class, helpful, ethnic, refugee

FREX: voted, euros2016, killed, working-class, helpful, ethnic, refugee

Lift: ethnic, refugee, euros2016, helpful, voted, killed, working-class

Score: voted, working-class, killed, euros2016, refugee, helpful, ethnic

Topic 8 Top Words:

Highest Prob: racist, voter, xenophobic, attacks, attack, brexiters, nations

FREX: racist, voter, xenophobic, attacks, attack, brexiters, nations

Lift: condemns, councillor, expats, attack, attacks, crime, elections

Score: racist, voter, attacks, xenophobic, attack, brexiters, nations

Topic 9 Top Words:

Highest Prob: britain, right, euro2016, work, economic, rights, workers

FREX: britain, right, euro2016, work, economic, rights, workers

Lift: britain, euromillions, original, economic, rights, work, campaigned

Score: britain, euro2016, right, work, economic, rights, workers

Topic 10 Top Words:

Highest Prob: vote, euro, france, nation, sadly, depressing, economics

FREX: vote, euro, france, nation, depressing, economics, helps

Lift: economics, vote, depressing, elect, euro, euroexit, france

Score: vote, euro, nation, france, euroexit, depressing, sadly

Topic 11 Top Words:

Highest Prob: country, voteremain, trump, business, english, war, businesses

FREX: country, voteremain, trump, business, english, war, businesses

Lift: borders, businesses, communities, culturalmarxism, europeanunion, muslims, nationalism

Score: country, voteremain, trump, business, english, newspapers, war

Topic 12 Top Words:

Highest Prob: £, voting, nhs, democracy, petition, scotland, votes

FREX: £, voting, nhs, democracy, votes, countries, protest

Lift: nationals, army, businessman, campaigner, democracy, economies, healthcare

Score: £, nhs, voting, democracy, votes, petition, scotland

Topic 13 Top Words:

Highest Prob: england, campaign, news, johnson, job, working, election

FREX: england, campaign, news, johnson, job, working, election

Lift: campaign, england, foreign, helped, job, news, newsnight

Score: england, campaign, news, johnson, job, minister, working

Topic 14 Top Words:

Highest Prob: european, tory, wales, enough, racism, french, $

FREX: european, tory, wales, enough, racism, french, $

Lift: brexiteers, criminal, depressed, europeans, eurotrash, foreigners, migrant

Score: $, european, tory, racism, enough, wales, french

Topic 15 Top Words:

Highest Prob: immigration, economy, iceland, border, right-wing, controls, eurostar

FREX: immigration, economy, iceland, border, right-wing, controls, eurostar

Lift: right-wing, border, immigration, economy, eurostar, iceland, sadday

Score: immigration, economy, iceland, border, eurostar, right-wing, controls
